# The Rationale for Monitoring Cognitive Function in Multiple Sclerosis: Practical Issues for Clinicians

**DOI:** 10.2174/1874205X01812010031

**Published:** 2018-05-31

**Authors:** Christos Bakirtzis, Panagiotis Ioannidis, Lambros Messinis, Grigorios Nasios, Elina Konstantinopoulou, Panagiotis Papathanasopoulos, Nikolaos Grigoriadis

**Affiliations:** 1 The Multiple Sclerosis Center, 2nd Department of Neurology, AHEPA University Hospital, Aristotle University of Thessaloniki, Thessaloniki, Greece; 2 Department of Neurology, Neuropsychology Section, University of Patras Medical School, Patras, Greece; 3 Department of Speech and Language Therapy, Higher Educational Institute of Epirus, Ioannina, Greece; 4 Lab of Cognitive Neuroscience, School of Psychology, Aristotle University of Thessaloniki, Thessaloniki, Greece; 5 University of Patras Medical School, Patras, Greece

**Keywords:** Multiple sclerosis, Cognition, Clinical practice, Monitoring, Depression, Fatigue

## Abstract

About half of patients with multiple sclerosis exhibit cognitive impairment which negatively affects their quality of life. The assessment of cognitive function in routine clinical practice is still undervalued, although various tools have been proposed for this reason. In this article, we describe the potential benefits of implementing cognitive assessment tools in routine follow -ups of MS patients. Early detection of changes in cognitive performance may provide evidence of disease activity, could unmask depression or medication side-effects and provide suitable candidates for cognitive rehabilitation. Since apathy and cognitive deficiencies are common presenting symptoms in Progressive Multifocal Leukoencephalopathy, we discuss the utility of frequent monitoring of mental status in multiple sclerosis patients at increased risk. In addition, we propose a relevant algorithm aiming to incorporate a systematic evaluation of cognitive function in every day clinical practice in multiple sclerosis.

## 1 INTRODUCTION

Multiple Sclerosis (MS) is a chronic demyelinating disease of the central nervous system. The relapsing -remitting form of the disease (RRMS) accounts for most of the cases, while the rest is characterized by progressive disability [1]. Disease symptoms in RRMS are mainly attributed to autoimmune inflammatory processes. However, it is now widely accepted that neurodegeneration occurs even in the early stages of MS and predominates in the progressive forms of the disease [2]. Beyond physical disability, there is increasing evidence that due to the same pathological processes, cognition is also affected and further contributes to the overall disability of the patient. Indeed, cognitive dysfunction can be detected in about half of MS patients and interferes with daily living activities [3].

In organized MS centers, cognitive evaluation is often performed by a neuropsychologist. In the setting of a private practice, on the other hand, cognitive assessment may be skipped due to limited time for consultancy. However, the American Academy of Neurology (AAN) underlines the fact that clinicians are often unable to detect cognitive deficits in everyday clinical practice [4] and emphasizes the importance of cognitive assessment as part of the everyday clinical examination - something that neurologists tend to forget or underestimate.

The Expanded Disability Status Scale (EDSS) is widely used in order to assess the neurological symptoms of an MS patient. In this scale, a sub score of cognitive function is included. The scoring depends on the clinician’s perception of cognitive functions of the patient. Also, according to the scoring manual, patients’ or caregivers’ opinion is also accounted while for precise scoring, a brief cognitive evaluation should be performed. A recent study has demonstrated that in a significant proportion of patients, the score of cognitive function in EDSS and subsequently the total EDDS score changes, after examination with a brief cognitive assessment battery [5]. Therefore, the use of assessment tools for cognitive function, could provide a more accurate evaluation of disease severity.

Cognitive deficits interfere with the ability to work, socialize and have a negative impact on quality of life in MS [6]. Memory deficits in MS are among the main causes of poor adherence to treatment [7, 8]. Hence, identifying them in the frame of neurological examination could be as useful as identifying other neurological symptoms for the holistic treatment of the patient.

## 2 WHAT TO MEASURE AND WHY?

Information processing speed (IPS), memory and to a lesser extent, executive function are the cognitive domains mainly affected in MS [6]. Although this pattern is frequently described, recent data suggest that cognitive dysfunction in MS might present in a more heterogeneous pattern amongst MS patients [9].

Cognitive impairment is often mild but may be profound at the late stages of the disease [10]; however, a number of patients may exhibit predominant cognitive deficits, despite minimal physical disability [11]. Persistent and progressive decline of cognitive performance is attributed to neurodegenerative processes of the disease such as diffuse axonal damage and brain atrophy [12]. In particular, deficits in IPS are mainly due to a «disconnection syndrome»; [13, 14] demyelinated axons are unable to transmit signals with high speed and neuronal degeneration leads to synaptic and dendritic loss, aggravating the deficit. Subsequently, MS patients face difficulties in everyday activities, particularly when the relevant involved tasks demand a heavy load of cognitive effort [15]. In this way, attention deficits are more obvious when multiple tasks are performed simultaneously, since patients get easily distracted [16]. Memory disturbances might be attributed to delayed retrieval secondary to the mechanisms described above [17], or to the damage of specific areas involved in memory storage [18].

Cognitive dysfunction in MS tends to progress during the disease course [19], but cognitive deterioration may also occur during relapses [20, 21]. According to experimental autoimmune encephalomyelitis (EAE) studies, this dysfunction may be attributed to the effect of cytokines released by inflammatory cells and activated microglia to neuron function [22]. The deterioration is often reversible during the recovery of the relapse [23]. Therefore, assessing cognitive function during a relapse could provide more information about the severity of the relapse and potentially alter the physician’s treatment plan. Interestingly enough, isolated cognitive relapses have been reported in the recent literature [24]; disease activity may be expressed with impairment of cognitive function without other new findings in routine clinical examination.

In addition, cognitive dysfunction may be profound during progressive multifocal encephalopathy (PML), an uncommon potentially fatal viral disease that might occur during treatment with some Disease Modifying Drugs (DMDs) [25]. Indeed, PML lesions may initially present in the frontal lobes, affecting cognition, mood and behavior of the patient, while other neurological symptoms may develop later [26, 27]. Importantly enough, cognitive evaluation in patients treated with DMDs with the potential incidence of PML may contribute to an earlier and safer identification of “subclinical” PML together with the use of MRI. Such a potential use of cognitive function is worth being studied since PML-related MRI findings may be miss-interpreted early enough to diagnose PML [28].

Mood disorders are commonly reported in MS and may contribute to the cognitive performance of MS patients. Mood swings are frequently observed, while depression seems to be a core feature of the disease [29]. Several studies indicate that depression in MS could be attributed to immune-mediated mechanisms together with dysfunction in the hypothalamic*–*pituitary*–*adrenal axis [30]. Suicidal rates are higher among MS patients [31]. A proportion of patients might present euphoric and inappropriate effect, exhibiting euphoria sclerotica [32]. Moreover, fatigue is a common symptom of the disease and has a physical and cognitive aspect. Both mood and fatigue can be confounding factors to patient’s cognitive performance, therefore they should also be assessed during a cognitive examination [33]. Diagnosis and treatment of mood disorders could be of benefit to the patient’s ability to cope with everyday life activities.

Benign MS accounts for 10-20% of cases depending on the study and definitions. A study of 163 patients with benign MS found that many of these patients presented cognitive impairment, depression and fatigue, questioning the term ‘’benign” MS [34]. These findings also suggest that with a more detailed clinical evaluation, hidden symptoms of MS may be unmasked. Cognitive assessment in everyday clinical practice could provide insight into the course of the disease. Patients with clinically isolated syndrome, presenting cognitive decline are more likely to convert to clinically definite multiple sclerosis [35]. Cognitive deficits in the early stages of the disease are predictors of a severe course of the disease [36]. In addition, there is an increasing number of uncommon MS phenotypes reported [37, 38], with predominant cognitive dysfunction as a presenting symptom. Therefore, a detailed evaluation of cognitive status of an MS patient could provide more insight to the disease burden, something that is still under-recognized.

## 3 HOW TO MEASURE?

A wide number of tools have been designed specifically for cognitive dysfunction in MS. Rao’s Brief Repeatable Battery (BRB) [39] and Minimal Assessment of Cognitive Function in Multiple Sclerosis (MACFIMS) [40] are the two most widely used batteries for the detection of cognitive impairment in MS. However, the administration of these batteries requires trained personnel and adequate amount of time [41]; therefore, their use in everyday clinical practice is limited. The Brief International Cognitive Assessment for Multiple Sclerosis (BICAMS) has been proposed as an easy, fast monitoring tool for use in everyday clinical practice [42]. This battery consists of three tests that examine different aspects of working memory and IPS. BICAMS requires no additional equipment and can be used as a bedside quick screening tool in clinical settings where neuropsychologists are not available. However, for a complete neuropsychological examination, patients should still undergo more comprehensive batteries [43].

Deficit in IPS is the most common finding in MS patients [44, 45] and is often undetected [46], therefore, at least the SDMT [47] should be performed in routine clinical examinations [41]. The test takes no more than five minutes to perform and can be repeated in reasonable time, since many equivalent alternate forms have been developed, thus minimizing practice effects [48]. In case of severe visual impairment, the Paced Auditory Serial Addition Test (PASAT) [49] could be administered instead. PASAT’s practice effects limit its use in longitudinal monitoring [50]. Current research provides evidence that besides IPS, working memory might influence the performance of an individual in both these tests [51, 52].

Importantly enough, mood and fatigue should also be assessed as both factors are confounders of the cognitive performance of an MS patient [53]. As for mood, there are a number of self-administered questionnaires such as The Beck Depression Inventory Second Edition (BDI-II) [54], Beck Depression Inventory-Fast Screen (BDI-FS) [55], Hospital Anxiety and Depression Scale (HADS) [56] amongst others. On the other hand, the tools most widely used to measure fatigue, mainly in clinical studies, are the Fatigue Severity Scale (FSS) and the Modified Fatigue Impact Scale (MFIS) [57]. All the above might be subject to an individual’s self-perception of mood or fatigue, while MS symptoms may be confounders of the performance [58]. Hence, in routine daily practice, depression and fatigue should also be assessed as per usual clinical care. Similarly, self-reported questionnaires about cognition in MS, might be useful in clinical studies but may not provide enough information about the cognitive domains affected to the examiner [59]. For example, the patient reported Multiple Sclerosis Neuropsychological Questionnaire (MSNQ-P) score may correlate with depression and anxiety but not with scores of cognitive tests [60, 61]. However, the informant version of the MSNQ (MSNQ-I) seems to describe more accurately the cognitive profile of an MS patient [62], thus indicating that it could be used as an alternative method for the preliminary evaluation of cognitive function in MS patients.

In the era of informatics, the development of computerized, fully automated cognitive tests, free from inter-rater variability, could provide even easier and more accurate measurements of cognitive function [63]. Tests like the Computerized Speed Cognitive Test (CSCT) [64], Global Assessment Battery [65] amongst others, have proven to be a reliable alternative to standard paper and pencil tests, however, they have not yet been widely adopted. Due to the increasing number of computerized cognitive batteries in development both the American Academy of Clinical Neuropsychology and the National Academy of Neuropsychology have released a position paper regarding the basic requirements needed to be fulfilled in order to produce an accurate and validated computerized cognitive battery [66].

Preservation of cognitive function is also depended on intellectual enrichment such as educational achievement and the lifestyle of each individual [67]. Therefore, cognitive reserve should also be taken into account, since an individual with a high education status and continuous cognitive exercise may cope with cognitive deficits even when there is a significant reduction of brain volume [68, 69]. Tools such as the Cognitive Reserve Index (CRIq) [70] for quantification of cognitive reserve could, therefore, be used.

Magnetic Resonance Imaging (MRI) markers have been proposed as an alternative to cognitive dysfunction measurements. Studies support that the rate of annual brain atrophy and the number of cortical lesions, can be predictors of deterioration of cognitive functions [71],while only a modest correlation has been found between T2 lesion load and cognitive impairment [72]. However, many imaging techniques are not standardized and brain atrophy measures still lack the accuracy needed for individual assessment in routine clinical practice [73]. The implementation of newer techniques such as Double Inversion Recovery, has allowed the imaging of cortical lesions, but compared to histopathological studies, not all of them are detected [74]. However, focusing on deep gray matter structures [75, 76] and using advanced structural and functional neuroimaging techniques might provide more insight regarding the anatomical routes of cognitive impairment in MS [77, 78]. Moreover, thalamic functional and structural changes have been extensively studied and were found to strongly correlate with cognitive impairment [76, 79, 80].

## 4 WHEN TO MEASURE AND HOW TO DEAL WITH FINDINGS?

The time interval between the cognitive assessment of a stable MS patient is under debate [81]. Both the British National Institute for Health and Care Excellence (NICE) [82] and AAN [4] suggest at least an annual routine assessment for these patients, but considering the cost, a fast screening by the treating physician could provide an alternative to patients who cannot afford a full neuropsychological evaluation. More frequent assessment should be reserved for patients with high PML risk or high disease activity, keeping in mind that practice effects may be augmented by frequent testing. An individualized approach considering the risk of cognitive deterioration, type of treatment, comorbidities and disease activity, should be tailored to each MS patient. Regarding relapses, as mentioned above, the cognitive evaluation could provide additional findings to support the neurological examination, as long as a previous cognitive status has been documented. Only by keeping archives of the cognitive examinations, can a physician detect cognitive deterioration regarding a previous state, something that patients sometimes report, but physicians find hard to evaluate [83].

Although no symptomatic treatment has provided adequate evidence for the treatment of cognitive dysfunction up to this point in time [84], preliminary data suggest a potential positive impact of fampridine in cognition and fatigue [85, 86]. Furthermore, research regarding non pharmaceutical cognitive rehabilitation interventions has provided indications that MS patients may benefit cognitively [87]. Questions about the required frequency of cognitive rehabilitation and the durability of the positive cognitive effects, however, remains to be answered by future studies. Functional MRI studies have also provided evidence for the efficacy of cognitive rehabilitation interventions, indicating that the activation of alternative cognitive pathways could compensate in part, for the cognitive deficits [88]. Adaptive neuronal plasticity occurs early in the disease course [89], therefore, monitoring the cognitive status of MS patients, might enable the early detection of cognitive impairment, providing suitable candidates for cognitive rehabilitation interventions.

Aerobic exercise may also ameliorate cognition, fatigue and mood of MS patients [90]. Clinicians should also advise patients to implement continuous intellectual enrichment [91] and avoid medications such as benzodiazepines that are known to negatively impact cognitive functions. But above all, treatment with DMDs that silence disease activity and slow the degeneration processes may be the main way to confront cognitive impairment in multiple sclerosis, at least based on evidence to this time point.

Taking into consideration that the use of the BICAMS is both time efficient and cost-effective for a screening evaluation of cognitive function in MS [41], it may be easily incorporated in everyday clinical practice. Most importantly, the identification of cognitive dysfunction may further unmask a number of potential factors, such as disease activity, fatigue, mood disturbances, drug interactions and side effects, which otherwise might have been disregarded. We therefore believe that it would be worth proposing an algorithm for clinician use as presented in Fig. (**1**), with tools that have been widely applied in MS studies and proven to have ecological validity, acknowledging the fact that new versions of tests or computer-based self-administered versions [92] might alter the near future routine cognitive testing of MS patients. The feasibility and cost-effectiveness of the proposed algorithm in every day clinical practice remains to be identified in prospective clinical studies.

Having mentioned all the above, we must acknowledge, that several issues still need to be resolved regarding the implementation of cognitive assessment in daily clinical practice. For example, there are no studies to support that the cognitive tests described are sensitive to PML. Cognitive reserve is built by experiences obtained not only by education but also by leisure activities [93], something difficult to quantify. Besides age and education, cognitive performance on specific tests might also be influenced by nationality [94], hence, development of universal norms for each test might be challenging. In addition, cognitive assessment performed in a ‘sterilized’ way, might not reflect cognitive issues of patients since they might perform well in tests highly specific to a single cognitive domain, but fail in multitasking real conditions [95]. Studies have shown that walking or other motor tasks might interfere with cognitive function in MS patients [96, 97], therefore dual-tasking measures may better reflect actual difficulties of MS patients [98].

## CONCLUSION

Cognitive dysfunction is common in multiple sclerosis and should be examined in routine clinical practice alongside with mood and fatigue. A detailed cognitive examination might reveal ‘hidden’ symptoms of the disease that may potentially alter treatment plans. Therefore, measures of cognitive function should be part of the routine neurological examination in MS patients. Guidelines on how to deal with cognitive deterioration in MS have not yet been produced and DMDs used in MS have only shown a minimal effect on cognitive function. But, to rephrase Lord Kelvin «*if you can’t measure it, you cannot improve it»* hence we could at least implement the cognitive examination in clinical practice.

## Figures and Tables

**Fig. (1):**
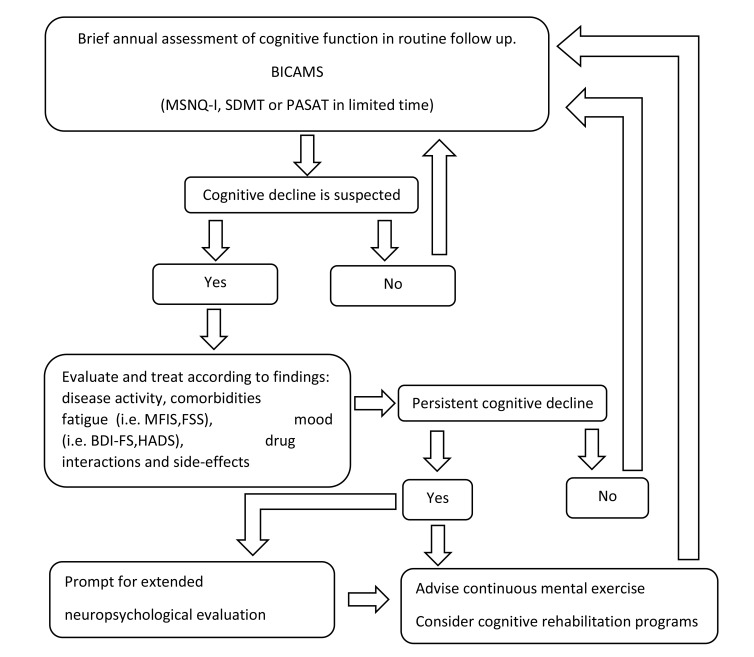
Proposed algorithm for the management of cognitive function in multiple sclerosis. *(BICAMS: Brief International Cognitive Assessment for Multiple Sclerosis, MSNQ-I: Multiple Sclerosis Neuropsychological Questionnaire Informant version, SDMT: Symbol Digit Modalities Test, PASAT: Paced Auditory Serial Addition Test, MFIS: Modified Fatigue Impact Scale, FSS: Fatigue Severity Scale, BDI-FS: Beck Depression Inventory-Fast Screen, HADS: Hospital Anxiety and Depression Scale*.
